# The in situ efficacy of whole room disinfection devices: a literature review with practical recommendations for implementation

**DOI:** 10.1186/s13756-022-01183-y

**Published:** 2022-12-05

**Authors:** Caroline M. van der Starre, Suzan A. J. Cremers-Pijpers, Carsten van Rossum, Edmée C. Bowles, Alma Tostmann

**Affiliations:** Unit of Hygiene and Infection Prevention, Department of Medical Microbiology, Radboud Center for Infectious Diseases (RCI), Radboudumc, Geert Grooteplein Zuid 10, 6525 GA Nijmegen, The Netherlands

**Keywords:** Whole room disinfection, Automated room disinfection, Aerosolized hydrogen peroxide, Hydrogen peroxide vapour, Ultraviolet C, Pulsed-xenon ultraviolet, Disinfection, Decontamination, Hospital acquired infections, Infection prevention and control

## Abstract

**Background:**

Terminal cleaning and disinfection of hospital patient rooms must be performed after discharge of a patient with a multidrug resistant micro-organism to eliminate pathogens from the environment. Terminal disinfection is often performed manually, which is prone to human errors and therefore poses an increased infection risk for the next patients. Automated whole room disinfection (WRD) replaces or adds on to the manual process of disinfection and can contribute to the quality of terminal disinfection. While the in vitro efficacy of WRD devices has been extensively investigated and reviewed, little is known about the in situ efficacy in a real-life hospital setting. In this review, we summarize available literature on the in situ efficacy of WRD devices in a hospital setting and compare findings to the in vitro efficacy of WRD devices. Moreover, we offer practical recommendations for the implementation of WRD devices.

**Methods:**

The in situ efficacy was summarized for four commonly used types of WRD devices: aerosolized hydrogen peroxide, H_2_O_2_ vapour, ultraviolet C and pulsed xenon ultraviolet. The in situ efficacy was based on environmental and clinical outcome measures. A systematic literature search was performed in PubMed in September 2021 to identify available literature. For each disinfection system, we summarized the available devices, practical information, in vitro efficacy and in situ efficacy.

**Results:**

In total, 54 articles were included. Articles reporting environmental outcomes of WRD devices had large variation in methodology, reported outcome measures, preparation of the patient room prior to environmental sampling, the location of sampling within the room and the moment of sampling. For the clinical outcome measures, all included articles reported the infection rate. Overall, these studies consistently showed that automated disinfection using any of the four types of WRD is effective in reducing environmental and clinical outcomes.

**Conclusion:**

Despite the large variation in the included studies, the four automated WRD systems are effective in reducing the amount of pathogens present in a hospital environment, which was also in line with conclusions from in vitro studies. Therefore, the assessment of what WRD device would be most suitable in a specific healthcare setting mostly depends on practical considerations.

**Supplementary Information:**

The online version contains supplementary material available at 10.1186/s13756-022-01183-y.

## Background

Hospitalized patients who have an infection with or are a carrier of a multidrug resistant micro-organism (MDRO), such as methicillin-resistant *Staphylococcus aureus* (MRSA), vancomycin-resistant Enterococcus (VRE) and multidrug resistant *Acinetobacter baumannii*, have to be cared for in isolation. After discharge of a patient with an MDRO, the patient room needs terminal cleaning and disinfection to eliminate the MDRO from the environment.

Terminal disinfection is often performed manually, which is labour intensive and prone to human errors. Errors can be made in the selection of the correct detergent, the formulation of the detergent in the correct concentration, the distribution of the product, and the adherence to the correct contact time [1–3]. This conventional way of disinfection therefore often does not eliminate all pathogens in a patient room [4–7]. Consequently, the risk for infection or carriage with the same MDRO for the next patient residing in such a patient room is at least two-fold but may even be greater than five-fold depending on the MDRO [8–10].

Automated ‘whole room disinfection’ (WRD) is a technology that can contribute to the quality of the terminal disinfection of patient rooms. WRD devices replace or add to the manual disinfection process by an automated process with a constant result. WRD still needs to be preceded by manual cleaning of the patient room, which is also common practice for manual disinfection.

Currently, there are four types of WRD devices that are used most frequently in a hospital setting. Aerosolized hydrogen peroxide (aHP) and H_2_O_2_ vapour make use of gaseous hydrogen peroxide. Ultraviolet C (UV-C) and pulsed-xenon UV (PX-UV) make use of ultraviolet radiation.

The efficacy of WRD devices is primarily assessed by its in vitro outcomes, which considers the logarithmic reductions of in vitro presence of pathogens after treatment with a WRD device. In vitro studies are performed in a controlled environment, where the investigated pathogen is cultured in specific quantities and treated with a standardized disinfection process. Therefore, in vitro evaluations may present the efficacy under ideal circumstances. Amongst the most frequently used types of WRD devices, H_2_O_2_ vapour has the highest in vitro efficacy, followed by UV-C.

In contrast to the in vitro conditions, the real-life settings in which WRD devices will be implemented, such as hospitals, present less optimal environments. The exact location and quantity of the pathogen is unknown and disinfection may be limited due to soiled surfaces. Additionally, WRD devices may not reach all areas of the patient room and efficacy may differ for the various kinds of surfaces. Furthermore, materials and medical equipment present in the room may not be suitable for or suffer from disinfection with e.g. UV-C or hydrogen peroxide. Therefore, the in situ efficacy may differ from the in vitro efficacy of WRD devices.

In this review we have summarized available scientific literature on the in situ efficacy of WRD devices in a hospital setting to get insight into the effect of WRD devices in real-life and less standardized settings. Therefore, we aim to describe the in situ efficacy of WRD devices in a hospital setting and how the in situ efficacy compares to the in vitro efficacy of such WRD devices. Moreover, we offer practical information concerning the WRD devices and provide recommendations for implementation.

## Methods

This review is limited to the four types of WRD devices that are most frequently used in a hospital setting: aHP, H_2_O_2_ vapour, UV-C and PX-UV. The efficacy of these WRD devices has been investigated for common micro-organisms known to cause hospital-acquired infections or hospital outbreaks, being norovirus, *Acinetobacter*, carbapenemase-producing Enterobacteriaceae (CPE), extended spectrum beta-lactamase (ESBL) producers, MRSA, VRE, *Clostridium difficile* and *Candida auris*.

In addition to a summary of the available literature concerning the efficacy of the four types of WRD devices, this review also describes and assesses the features and practicalities of each type. Data was collected via literature and through interviews with experts in hygiene and infection prevention, both within our unit as from other Dutch hospitals.

### Outcome measures

For in situ efficacy, a distinction was made between environmental and clinical outcome measures. The environmental outcome measure was defined as the difference in the microbial contamination before and after disinfection with a WRD device, expressed in the number of positively tested sites or rooms, or the number of colony-forming units (CFUs) sampled in a patient room. This difference was converted to a percentage (increase or reduction in micro-organism). When both data regarding positive sites and rooms were reported in an article, data concerning positive sites were favoured as this information is more detailed. Only when no data concerning positive sites was available, data concerning the number of positive rooms was reported. The effect of disinfection as regarded in the studies performed for a certain micro-organism was summarized in a range of effect. In this range, data regarding the differences in the number of positive sites, rooms and CFUs were combined.

The clinical outcome measures was defined as the difference between the pre- and post-implementation period patient infection rate. This difference was converted to a percentage of increase or reduction. Both the microbial colonization or acquisition infection rates and the clinical infection rates were included in the clinical outcome measure.

The in vitro efficacy of the four WDR systems for the predefined micro-organisms was determined using a non-systematic literature search.

### Search strategy

A literature search was performed in the medical database PubMed in September 2021. After the initial search, it was discovered that the terms “patient” and “patient room” yielded relevant articles as well. A second literature search was carried out with an adjusted search string. The initial and second literature search are presented in Table 1. Articles were primarily selected based on title and abstract. Subsequently, a final selection based on relevance of the full text was made.

**Table 1:** The initial and second search string as applied in PubMed in September 2021

	(“Acinetobacter“[tiab] OR “Norovirus“[tiab] OR “NoV“[tiab] OR “VRE“[tiab] OR “Vancomycin-resistant enterococc*“[tiab] OR “CPE[tiab]” OR “Carbapenemase-Producing Enterobacteral*“[tiab] OR “MRSA“[tiab] OR “methicillin resistant *Staphylococcus aureus*“[tiab] OR “ESBL“[tiab] OR “Extended spectrum beta-lactamase“[tiab] OR “Candida Auris“[tiab] OR “C. auris“[tiab] OR “clostridium difficile“[tiab] OR “C. Difficile“[tiab] OR “Ebolavirus“[tiab] OR “Lassa Virus“[tiab] OR “Marburgvirus“[tiab])
AND	(“VHP“[tiab] OR “vaporized hydrogen peroxide“[tiab] OR “HPV“[tiab] OR “hydrogen peroxide vapor“[tiab] OR “aHP“[tiab] OR “aerosolized hydrogen peroxide“[tiab] OR “hydrogen peroxide vapour“[tiab] OR “PX-UV“[tiab] OR “pulsed xenon UV“[tiab] OR “UV-C“[tiab] OR “ultraviolet C“[tiab] OR “no-touch disinfection”[tiab] OR “whole room disinfection”[tiab] OR “automated room disinfection”[tiab])
AND 1st search string	(“hospital” [tiab] OR “ward” [tiab] OR “care institution” [tiab] OR “emergency room” [tiab] OR “facility” [tiab] OR “clinic” [tiab] OR “medical centre” [tiab] OR “nursing home” [tiab] OR “insitution” [tiab] OR “health centre” [tiab] OR “infirmary” [tiab])
2nd search string	(“patient” [tiab] OR “patient room” [tiab])

### Inclusion and exclusion criteria

Articles were applicable for inclusion if (1) they focussed on the decontamination of a (patient) room after discharge of a patient with one of the predefined micro-organisms; (2) the automated room disinfection was performed using aHP, H_2_O_2_ vapour, UV-C or PX-UV; (3) it was published between January 2000 and September 2021 in a medical scientific journal; (4) it was available in English language; (5) it reported at least one of the environmental or clinical outcome measures of interest; and (6) applied WRD devices as a solitary intervention. Articles describing infections with micro-organisms other than those previously predefined or articles that did not specify included micro-organisms and articles that studied the effect of a bundle of interventions instead of the solitary effect of WRD were excluded from this review. The preparation of a patient room by cleaning (and decontamination) prior to disinfection with WRD were not regarded as a bundle of interventions, as cleaning is a crucial preparational element of WRD.

### Data extraction

Data extracted from the included articles were: name of authors, year of publication, country in which the study was performed, setting (i.e. type of care institution in which the study was performed), study design, associated pathogen, type of WRD device and environmental or clinical outcome measure.

For each of the four types of WRD devices, a detailed description of the features, practicalities, in vitro and in situ efficacy is provided.

## Results

### Article selection

**Fig. 1:**
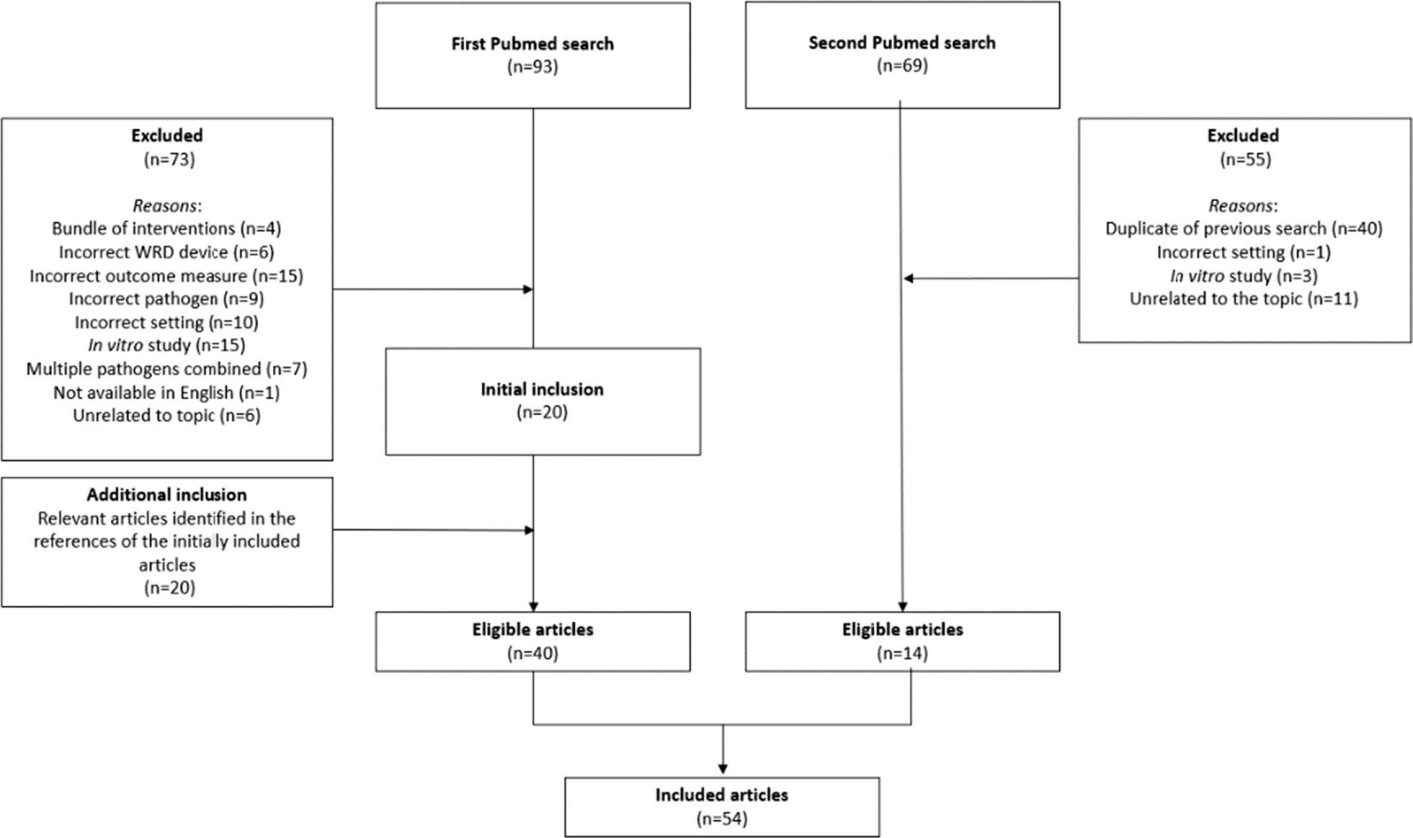
Flow diagram of the two literature searches and the article selection

The initial search string yielded a total of 93 articles. After assessing these articles for eligibility, 73 articles were excluded. The main reasons for exclusion were having an incorrect outcome measure, an incorrect (or not hospital-related) setting or assessing in vitro instead of in situ efficacy. Through the assessment of references mentioned by the 20 eligible articles, another 20 relevant articles were included into the first literature search. A second search string yielded a total of 69 articles, of which 14 were eligible for inclusion. Based on the initial and second PubMed searches, a total of 54 articles regarding the in situ efficacy of WRD devices were included into the review. A flowchart of the article selection process is presented in Fig. 1.

### General findings

There was a large variation between the included studies in methodology, reported outcome measures, preparation of the patient room prior to environmental sampling, the location of sampling within the room and the moment of sampling. This means that the findings were sometimes difficult to summarize. However, when the direction or magnitude of the effect was similar, outcomes have been combined when possible.

In articles reporting environmental outcome measures, the sites that were sampled within a room also differed per study and varied from high-touch surfaces to other specific surfaces within a patient room such as medical equipment or furniture. Moreover, the preparation prior to sampling differs between studies. In most studies the patient room was cleaned before disinfection, however some studies implemented WRD systems in an uncleaned room. The reported timing of sampling also varied from sampling before cleaning and after disinfection to sampling after cleaning and after disinfection to a combination of this.

Studies regarding clinical outcomes also varied in the reported outcome measures. The majority of the included articles reported the microbial colonization (or acquired) infection rate (n = 14). Others reported the clinical infection rate (n = 4) or did not specify the infection rate (n = 1).

Additional file 1: Tables S1, S2, S3, S4, S5, S6, S7 and S8 show a complete overview of the included articles and their main outcomes .

### Aerosolized hydrogen peroxide

#### Features

Aerosolized hydrogen peroxide (aHP) is a type of WRD in which a solution of 5–6% hydrogen peroxide is fogged into a patient room. During this process of fogging, a so-called ‘dry mist’ is formed which spreads through patient room and disinfects the contact surfaces [11]. Following exposure, hydrogen peroxide (HP) is naturally broken down to water and oxygen.

#### Practicalities

Automated room disinfection with aerosolized hydrogen peroxide has several benefits and limitations affecting the in situ efficacy. A benefit of aHP is that it is user friendly. The machine consists of only one unit and is therefore easy to transport within a hospital. Preparations of the patient room include cleaning and enlarging of the contact area. Although manufacturers state that the sealing of vents and doors with tape is not necessary, sealing is recommended by literature to prevent leakage of HP, which makes the preparations more labour intensive [12]. Furthermore, aHP is capable of disinfecting difficult-to-reach areas, such as the inside of a drawer or the back of a closet. However, aerosolized hydrogen peroxide particles are affected by gravity and aHP devices often distribute HP only in one direction due to the unidirectional nozzle [11]. Consequently, the distribution of aerosolized hydrogen peroxide in a room is sometimes not homogeneous.

A limitation of aerosolized hydrogen peroxide is the duration of disinfection. Hydrogen peroxide is toxic and therefore no people are allowed in the room during disinfection. A room that is disinfected with hydrogen peroxide may be re-entered only if the concentration of hydrogen peroxide has declined to 1 parts per million (ppm). The so-called aeration phase, the time between the injection of hydrogen peroxide in a room and the moment the room can be re-entered, is the main reason for the lengthy cycle time. For a single patient room, the cycle time is approximately 2–3 h [13–16]. Moreover, sometimes the recommended ppm’s are not achieved rendering the performed procedure less or not effective. Similar to all other WRD devices, the efficacy of hydrogen peroxide is limited when a micro-organism is dissolved in (organic) material. Therefore, rooms must be cleaned (manually) before disinfection [12, 14, 15]. Compatibility of aHP with hospital materials has not been investigated thoroughly, but aHP seems compatible with metals, plastics and other materials.

#### In vitro efficacy

Ten articles have been included to assess the in vitro efficacy of aHP regarding the preselected micro-organisms (Table 2) [12, 14, 16–23]. For norovirus, the estimated reduction is 2.5-log for the virus itself and 4.5-log for its surrogate (Table 2) [18–20]. The in vitro efficacy regarding bacteria shows a large variation, with relatively low log-reductions of 1–1.7 for VRE and *Acinetobacter* respectively, and higher reductions for MRSA (> 4-log) and ESBL (> 6-log) [12, 14, 16, 17, 21]. *C. difficile* shows a reduction of ~ 5-log [12, 14, 22, 23]. No research has been performed regarding aHP efficacy for CPE or *C. auris*.

**Table 2:** In vitro efficacy of aHP for the preselected set of micro-organisms, expressed in log-reduction

	Micro-organism	Effect in log_10_ reduction, median (range)	N (ref)
Viruses	Norovirus (Surrogate)	2.5 (0.5–2.7) 4.5 (> 4–5.3)	3 [12, 18, 19] 3 [18–20]
Bacteria	*Acinetobacter*	2 (1–>4)	2 [12, 16]
	CPE		
	VRE	1–1.7	1 [21]
	ESBL	> 6	1 [14]
	MRSA	> 4 (2–>6)	4 [12, 14, 16, 17]
Spores	*C. difficile*	4.9 (0.13–>5)	4 [12, 14, 22, 23]
Yeast	*C. auris*		

#### In situ efficacy 

For the assessment of in situ efficacy of aHP, nine articles have been included [15, 17, 24–30].

##### Environmental outcomes

Environmental outcomes were described for *Acinetobacter*, MRSA, VRE and *C. difficile* (Table 3). All studies except for two reported the number of positive sites as an outcome measure. All studies observed a reduction in microbioal contamination after disinfecting with aHP. Statistical significance was reported in only three out of the nine articles [15, 28, 30]. For A*cinetobacter*, a statistically non-significant 78–100% reduction in bacterial load was observed in two studies [24, 25]. Efficacy of aHP against MRSA ranged from a 10–100% reduction [17, 26–28]. Only one study reported significance and observed a statistically significant reduction in MRSA load of 23.8% [28]. One study investigated the efficacy of aHP against VRE and observed a complete reduction in bacterial load [26] This study did not report significance. Lastly, a 59.4–94% reduction was observed for *C. difficile* after exposure to aHP. This range specifies to a reduction of 87.5–91% when merely observing the statistically significant outcomes [15, 29, 30]. No in situ studies were performed regarding norovirus, CPE, ESBL, and *Candida auris.*

##### Clinical outcomes

Clinical efficacy of aHP was assessed for one study only (Table 3). Herein, a 41.1% (*p* < 0.001) reduction in hospital MRSA infection rate was observed after implementation of aHP following manual cleaning [28]. There were no in situ studies reporting hospital infection rates for the other pathogens.

**Table 3:** An overview of the environmental and clinical outcomes of studies using aerosolized hydrogen peroxide

		Environmental outcomes			Clinical outcomes		
	Micro-organism	Range of effect of all studies	Range of effect of stat. sign. findings	Number of studies	Range of effect of all studies	Range of effect of stat. sign. findings	Number of studies
Viruses	Norovirus						
Bacteria	*Acinetobacter*	78–100% reduction		2 [24, 25]			
	ESBL						
	CPE						
	MRSA	10–100% reduction	23.8% reduction	4 [17, 26–28]	41.1% reduction	41.1% reduction	1 [28]
	VRE	100% reduction		1 [26]			
Spores	*C. difficile*	59.4–94% reduction	87.5–91% reduction	3 [15, 29, 30]			
Yeast	*C. auris*						

### H_2_O_2_ vapour

#### Features

Systems using H_2_O_2_ vapour evaporate a 30–35% hydrogen peroxide solution into a (patient) room. The hydrogen peroxide is broken down to water and oxygen after exposure. This decomposition is often facilitated by a aeration unit which reduces the disinfection cycle time [11].

#### Practicalities

Similar to aHP, a benefit of H_2_O_2_ vapour is its ability to disinfect difficult-to-reach areas. Moreover, due to heat-generated evaporation of hydrogen peroxide and the presence of multiple nozzles on the devices, the H_2_O_2_ vapour is homogenously distributed amongst the patient room [11].

H_2_O_2_ vapour systems often consist of multiple units and are less straightforward in use than aHP systems. Moreover, the preparations before disinfection are time-consuming and labour intensive. To prevent leakage of hydrogen peroxide, vents and entry doors must be sealed off. Disinfection with H_2_O_2_ vapour is time consuming. No people may remain in the room during disinfection and the patient room may only be re-entered if the concentration of hydrogen peroxide has declined under the health and safety exposure limit of 1 ppm. Due to the active aeration unit, the cycle time (the disinfection cycle excluding preparations) is somewhat shorter than that of aHP and is estimated at 1.5–2 h for a single patient room [13, 31–41]. Although most materials in a patient room are compatible with hydrogen peroxide, some materials or objects (e.g. nylon) can be damaged.

#### In vitro efficacy

H_2_O_2_ vapour has been shown to effectively reduce norovirus in in vitro settings, in which log reductions of > 4-log have been observed (Table 4) [18, 33, 34, 42, 43]. For bacteria, reductions of > 5-log were observed for A*cinetobacter* and of > 6-log for CPE, VRE and MRSA [12, 14, 35, 36, 44–47]. Similarly, > 6-log reductions were also observed for *C. difficile* [12, 23, 31, 37, 44, 48]. No studies are performed regarding the in vitro efficacy of ESBL and *C. auris*.

**Table 4:** In vitro efficacy of H_2_O_2_ vapour for the preselected set of micro-organisms, expressed in log-reduction

	Micro-organism	Effect in log_10_ reduction, median (range)	N (ref)
Viruses	NoV (Surrogaat)	> 4 4.4 (3–≥6)	1 [18] 4 [33, 34, 42, 43]
Bacteria	*Acinetobacter*	> 5 (> 4–>6)	5 [12, 14, 35, 44, 45]
	CPE	> 6	2 [44, 45]
	VRE	> 6 (> 4–>6)	3 [35, 44, 45]
	ESBL		
	MRSA	> 6 (3–>6)	7 [12, 14, 35, 36, 44, 46, 47]
Spores	*C. difficile*	> 6 (> 5,7–>6)	6 [12, 23, 31, 37, 44, 48]
Yeasts	*C. auris*		

#### In situ efficacy

The in situ efficacy of H_2_O_2_ vapour has been assessed in a total of 11 articles [4, 6, 39–41, 49–54].

##### Environmental outcomes

Environmental outcomes were described for *Acinetobacter*, MRSA, VRE and *C. difficile* (Table 5) [4, 6, 41, 49–54]. Three studies reported only number of positive rooms as an outcome measure, the others reported number of positive sites. Statistical significance was reported in only three studies [6, 41, 54]. For A*cinetobacter*, a 73–100% reduction in microbial load was observed after disinfection [6, 49]. Only the study reporting a 73% reduction described statistical significance [6]. Six studies regarding MRSA observed a range of effect from an increase of 11.1% to a reduction in contamination of 100% [4, 6, 50–53]. Only two of these studies reported significance [6, 41], of which one described a statistically significant reduction of 98.1% in MRSA contamination [6]. For VRE, a non-statistically significant reduction in bacterial load ranging from 6.4 to 100% was observed [41, 50]. Lastly, two studies observing *C. difficile* both reported a complete reduction in bacterial load after disinfection with H_2_O_2_ vapour [41, 54]. One of these studies was statistically significant [54]. No in situ studies regarding environmental outcomes were performed for norovirus, CPE, ESBL and *C. auris.*

##### Clinical outcomes

Clinical efficacy of H_2_O_2_ vapour was assessed in four studies for MRSA, VRE and *C. difficile* (Table 5) [39–41, 54]. All studies reported a decrease in incidence rate. A non-significant reduction in MRSA infection rate was observed of 68%. The infection rate of VRE reduced significantly by 79.3% in a study by Passaretti et al. [41]. The range of effect of all studies for *C. difficile* was a reduction of 37–63%. The statistical range of effect was approximately similar with a 37–60% reduction. No studies regarding the infection rate were performed for norovirus, *Acinetobacter*, CPE, ESBL and *C. auris.*

**Table 5:** An overview of the environmental and clinical outcomes of studies using hydrogen peroxide vapour

		Environmental outcomes			Clinical outcomes		
	Micro-organism	Range of effect of all studies	Range of effect of stat. sign. findings	Number of studies	Range of effect of all studies	Range of effect of stat. sign. findings	Number of studies
Viruses	Norovirus						
Bacteria	*Acinetobacter*	73–100% reduction	73% reduction	2 [6, 49]			
	CPE						
	ESBL						
	MRSA	11.1% increase to 100% reduction	98.1% reduction	7 [4, 6, 41, 50–53]	68% reduction		1 [41]
	VRE	6.4–100% reduction		2 [41, 50]	79.3% reduction	79.3% reduction	1 [41]
Spores	*C. difficile*	100% reduction	100% reduction	2 [41, 54]	37–63% reduction	37–60% reduction	4 [39–41, 54]
Yeast	*C. auris*						

### Ultraviolet C

#### Features

Systems making use of ultraviolet C (UV-C) emit a radiation with a wavelength of 254 nm. This radiation is constantly emitted during the disinfection cycle. This radiation disrupts the stability of nucleic acids, which is fatal for a cell due to consequences on metabolism and cell division [55, 56].

A broad range of UV-C systems are currently offered on the market. A distinction can be made between stationary systems and moving systems. Stationary systems have to be moved within the patient rooms by an operator in-between disinfection cycles to disinfect all areas. On the contrary, more advanced systems move autonomously through a patient room.

#### Practicalities

Automated room disinfection with ultraviolet radiation has several benefits and limitation. A benefit of disinfection with UV-C is the short disinfection time. In contrast with hydrogen peroxide, a room is immediately accessible after disinfection is complete as no aeration is needed. Disinfection of a single patient room with a stationary device is estimated at 50 min, while a moving robot takes 10–20 min [57, 58]. The room must be emptied of people during decontamination as exposure to UV radiation is not safe for humans. Moreover, UV-C disinfection does not make use of chemical products and leaves no residue rendering the process more environmental friendly [59].

A limitation of UV-C is that the efficacy of disinfection is reduced in areas that are out of the direct line of sight. UV-C is most effective when a micro-organism is directly exposed to the radiation. If a micro-organism is shaded from direct UV radiation, e.g. due to an obstruction or its inaccessible location, efficacy is limited [59]. Moreover, UV-C efficacy is reduced when the distance to an object that is to be disinfected increases or when the exposure time is limited [57, 60, 61]. To conclude, objects in a patient room containing polymers (i.e. medical devices and consumables) are susceptible to UV radiation and might be damaged by this type of WRD system.

#### In vitro efficacy

UV-C elicits log reductions of ≥ 4 for all predefined bacteria under optimal conditions (Table 6) [58, 60–72]. ESBL is the most susceptible, where log reductions of > 8 have been shown [62, 65]. In vitro efficacy under suboptimal conditions is greatly reduced, with most bacteria showing reductions of approximately 3-log. For *C. difficile*, a reduction of 2.5-log is observed under optimal, and a < 2-log reduction under suboptimal circumstances [31, 57, 60, 61, 66–72]. For *C. auris*, the optimal log reduction is > 5 and the suboptimal 3.3-log [72–75]. No studies were performed regarding norovirus.

**Table 6:** In vitro efficacy of UV-C for a preselected set of micro-organisms under optimal and suboptimal circumstances

	Micro-organism	Optimal circumstances; effect in log_10_ reduction, median (range)	Suboptimal circumstances; effect in log_10_ reduction, median (range)	N (ref)
Viruses	Norovirus			
Bacteria	*Acinetobacter*	≥ 4 (≥ 4–>8)	3 (< 1–4)	6 [58, 61–65]
	CPE	4–5	1–5	1 [66]
	ESBL	> 8	> 3	2 [62, 65]
	MRSA	4 (2–9)	< 3 (< 1–>6)	13 [58, 60–63, 65–72]
	VRE	3.9 (2–>8)	< 3 (< 1–>4)	10 [58, 60–63, 67–71]
Spores	*C. difficile*	2.5 (1–>5)	< 2 (0–>3)	11 [31, 57, 60, 61, 66–72]
Yeasts	*C. auris*	> 5 (3.99–>6)	3.3 (< 2–>4)	4 [72–75]

Under suboptimal circumstances, the investigated micro-organism is diluted in organic soil, the sample does not receive direct UV-C light, received UV-C radiation for only a limited time or is placed at a further distance from the device. The in vitro efficacy is expressed in log reduction.

#### In situ efficacy

A total of 13 articles was published regarding the in situ efficacy of UV-C, of which 8 focused on environmental outcomes [60–62, 76–80] and 5 on clinical outcomes [81–85].

##### Environmental outcomes

Two studies reported CFU as the outcome measure, one study reported the number of positive rooms and five studies reported the number of positive sites. All articles concerning the environmental outcomes reported a reduction in microbial contamination after disinfection (Table 7). For A*cinetobacter*, a total reduction of 35.8–98% in microbial load was observed [76, 77]. The reduction of 98% was statistically significant [77]. For MRSA, the range of effect of all studies ranged between a reduction of 66.7–100% and the statistically significant range between a 79–97.5% reduction [60–62, 77–79]. For VRE, a reduction of 21.8–100% in microbial contamination was reported [60–62, 76–78] the statistically significant range was considerably smaller with a 83.3–100% reduction. The range of effect of all studies associated with *C. difficile* ranged from a 23.9–100% reduction [60, 76–80]. The statistically significant range of effect was ranged between an 80–100% reduction. Only one study was performed regarding *C. auris* [79]. However, all *C. auris* had already been removed after the initial cleaning step, rendering additional disinfection unnecessary. No studies regarding the environmental decontamination were performed for norovirus, CPE and ESBL.

##### Clinical outcomes

Large discrepancies between micro-organisms were observed in the assessment of clinical outcomes related to disinfection with UV-C (Table 7). The ranges of effect of all studies observed both increases and decreases in infection rate, whilst the statistically significant ranges of effect only reported decreases. For *Acinetobacter* and VRE, the total ranges of effect were similar to the significant ones and described respectively a 53.1–71.8% and a 0.3–33.8% reduction in infection rates. For CPE, the range of effect of all studies ran from a 7.5% increase to a 100% decrease [82, 83]. Only the study reporting a 100% decrease in infection rate was found statistically significant [82]. One study only reported the change in infection rate of ESBL after UV-C disinfection, which showed a non-significant 12.9% reduction [82]. Lastly, the range of effect of all studies for *C. difficile* ran from a 26.1% increase to a 46.2% decrease [80, 81, 83–85]. The statistically significant range only showed reductions in infection rate ranging between 9.6 and 46.2%. No studies regarding the infection rate were performed for norovirus and *C. auris.*

**Table 7:** An overview of the environmental and clinical outcomes of studies using UV-C

		Environmental outcomes			Clinical outcomes		
	Micro-organism	Range of effect of all studies	Range of effect of stat. sign. findings	Number of studies	Range of effect of all studies	Range of effect of stat. sign. findings	Number of studies
Viruses	Norovirus						
Bacteria	*Acinetobacter*	35.8–98% reduction	98% reduction	2 [76, 77]	53.1–71.8% reduction	53.1–71.8% reduction	3 [81–83]
	CPE				7.5% increase to100% reduction	100% reduction	2 [82, 83]
	ESBL				12.9% reduction		1 [82]
	MRSA	66.7–100% reduction	79–97.5% reduction	6 [60–62, 77–79]	9.5% increase to 30.8% reduction	30.8% reduction	3 [81–83]
	VRE	21.8–100% reduction	83.3–100% reduction	6 [60–62, 76–78]	0.3–33.8% reduction	0.3–33.8% reduction	3 [81–83]
Spores	*C. difficile*	23.9–100% reduction	80–100% reduction	6 [60, 76–80]	26.1 increase to 46.2% reduction	9.6–46.2% reduction	5 [80, 81, 83–85]
Yeast	*C. auris*	100% reduction after cleaning	100% reduction after cleaning	1 [79]			

### Pulsed-xenon UV

#### Features

Pulsed xenon ultraviolet (PX-UV) also make use of ultraviolet radiation. As opposed to UV-C, which only emits radiation at a constant wavelength, PX-UV emits radiation of a broad spectrum of wavelengths (200–320 nm). This spectrum includes both UV-C, UV-B and UV-A radiation. Moreover, the radiation is not emitted continuously, but with short pulses [69, 86, 87].

#### Practicalities

The benefits and limitations of PX-UV are similar to those of UV-C devices. Disinfection of a single patient room with PX-UV, including the manual repositioning of the stationary device, only takes approximately 12–20 min [88–97]. Moreover, PX-UV devices are environmental friendly as no chemicals are used in the disinfection process and no residue is left.

Similar to UV-C, the main limitation of PX-UV is the limitation of its efficacy due to shading [68, 69, 88]. Other factors limiting the efficacy of UV are an increased distance between the device and the surface and a shortened disinfection time [69, 88, 98]. Moreover, as the device is stationary, it has to be repositioned by an operator during the disinfection cycle.

#### In vitro efficacy

There is considerable variety in efficacy of PX-UV between the predefined micro-organisms (Table 8). *Acinetobacter* and ESBL-producing bacteria are very susceptible to PX-UV, log reductions of > 5 have been observed under optimal circumstances [88]. On the contrary, MRSA and VRE show reduced susceptibility to PX-UV. Log reductions of 3.3 for MRSA and 2.7 for VRE have been reported previously under optimal circumstances [68, 69, 88]. Susceptibility is even further reduced when assessing efficacy under suboptimal circumstances. The reported log reductions were approximately two-fold lower for MRSA (1.5-log) and four-fold lower for VRE (0.1–0.6-log). *C. difficile* shows a log reduction of 1.8 under optimal conditions, and 0.7 under suboptimal circumstances [68, 69, 88]. The in vitro log reduction for *C. auris* is solely investigated under optimal circumstances, being 0.04–1.19-log [68, 69, 88]. No data in vitro data was available for norovirus and CPE.

**Table 8:** In vitro efficacy of PX-UV for a preselected set of micro-organisms, expressed in log_10_ reduction

	Micro-organism	Optimal circumstances; effect in log_10_ reduction, median (range)	Suboptimal circumstances; effect in log_10_ reduction, median (range)	N (ref)
Viruses	Norovirus			
Bacteria	*Acinetobacter*	> 5		1 [88]
	CPE			
	ESBL	> 5		1 [88]
	MRSA	3.3 (< 2–>5)	1.5 (0.7–1.9)	3 [68, 69, 88]
	VRE	2.7 (< 2–>5)	0.1–0.6	3 [68, 69, 88]
Spores	*C. difficile*	1.8 (< 1–3,4)	0.7 (0.2–0.8)	3 [68, 69, 88]
Yeasts	*C. auris*	0.04–1.19		2 [98, 99]

#### In situ efficacy

A total of 20 articles were published regarding the in situ efficacy of PX-UV, of which 10 focused on environmental outcomes and 10 on clinical outcomes.

##### Environmental outcomes

In situ efficacy of PX-UV regarding environmental outcomes has only been investigated for three of the preselected micro-organisms, being MRSA, VRE and *C. difficile* (Table 9). Five articles reported CFU/colonies as the outcome measure, the rest reported number of positive sites. All of the included articles observed a reduction in microbial contamination after disinfection. For MRSA, both total and statistically significant effect ranged from a 72.7–90.9% reduction in microbial contamination [69, 97, 100–102]. The statistically significant range of effect for VRE was considerably smaller than the total range of effect, respectively 38–100% reduction versus 75–100% reduction in contamination [69, 91, 103, 104]. For *C. difficile*, the total and statistically range of effect did not differ considerably, respectively 59–100% and 63.6–94.9% reduction in microbial contamination [69, 105, 106]. No studies were performed for norovirus, A*cinetobacter*, CPE, ESBL, and *C. auris.*

##### Clinical outcomes

The ranges of effect of all studies for the preselected micro-organisms observed both increases and decreases in infection rate, whilst the statistically significant ranges of effect only reported decreases (Table 9). For *Acinetobacter*, a statistically significant reduction of 63% in infection rate was regarded [89]. For MRSA, the range of effect of all studies ran from a 20% increase in infection rate to a 37.9% decrease [89, 92, 95, 107]. The statistically significant range only observed reductions in infection rate ranging from 26.7 to 37.9%. For VRE, that total range of effect ran from an increase of 18.9% to a decrease of 52.7%, while the statistically significant range ran from a 18.9% reduction to a 50% reduction [90, 92, 93, 107]. Lastly, the total *C. difficile* range also observed both increases and decreases in infection rate [85, 90, 92–94, 96, 107, 108]. The statistically significant range merely observed reductions ranging from 17.2 to 53%. No studies were performed for norovirus, CPE, ESBL and *C. auris.*

**Table 9:** An overview of the environmental and clinical outcomes of studies using PX-UV

		Environmental outcomes			Clinical outcomes		
	Micro-organism	Range of effect of all studies	Range of effect of stat. sign. findings	Number of studies	Range of effect of all studies	Range of effect of stat. sign. findings	Number of studies
Viruses	Norovirus						
Bacteria	*Acinetobacter*				63% reduction	63% reduction	1 [89]
	CPE						
	ESBL						
	MRSA	72.7– 90.9% reduction	72.7–90.9% reduction	5 [69, 97, 100–102]	20% increase to 37.9% reduction	26.7–37.9% reduction	4 [89, 92, 95, 107]
	VRE	38–100% reduction	75–100% reduction	4 [69, 91, 103, 104]	18.9% increase to 52.7%	18.9–50% reduction	4 [90, 92, 93, 107]
Spores	*C. difficile*	59–100% reduction	63.6–94.8% reduction	3 [69, 105, 106]	18.6% increase to 53% reduction	17.2–53% reduction	8 [85, 90, 92–94, 96, 107, 108]
Yeast	*C. auris*						

.

### Comparison of WRD types

In the previous section of the results, we have presented the practical considerations, in vitro efficacy and in situ efficacy for the 4 typed of WRD devices. In Table 10, we show a side-by-side comparison of these data. The comparison must be regarded subjectively, as the outcomes do not stem from one article or source. More detailed information concerning the characteristics of the WRD devices can be found in the previous result sections.

**Table 10:** Comparison of the characteristics of aHP, H_2_O_2_ vapour, UV-C and PX-UV devices

	aHP	H_2_O_2_ vapour	UV-C	PX-UV
In vitro efficacy	Viruses: 2.5-log Bacteria: >1 - >6-log Spores 4.9-log Fungi: –	Viruses: >4-log Bacteria: >5 - >6-log Spores > 6-log Fungi: –	Viruses: – Bacteria: >3.9 - >8-log Spores 2.5-log Fungi: >5-log	Viruses: – Bacteria: >2.7 - >5-log Spores 1.8-log Fungi: 1-log
In situ efficacy	Reduction of environmental and clinical outcomes	Reduction of environmental and clinical outcomes	Reduction of environmental and clinical outcomes	Reduction of environmental and clinical outcomes
Homogeneity of disinfection	Not homogenous, unidirectional	Homogenous	Not homogenous under suboptimal circumstances	Not homogenous under suboptimal circumstances
Prior preparations in patient room	Cleaning, turning off smoke alarms	Cleaning, sealing doors/vents, turning off smoke alarms	Cleaning, removing curtains	Cleaning, removing curtains
Duration disinfection	2–3 h	1.5–2 h	Moving robot: 10–20 min Stationary robot: 50 min	12–20 min
Required equipment	Device + disinfecting substance	Device + disinfecting substance	Device only	Device only
Compatibility materials	Not extensively investigated	Compatible with most materials	Might damage polymers	Not extensively investigated

## Discussion

In this review, we summarized available literature about the in situ efficacy of four types of whole room disinfection devices, aerosolized hydrogen peroxide, H_2_O_2_ vapour, ultraviolet C and pulsed xenon ultraviolet, and assessed the differences with in vitro efficacy. In situ efficacy was determined by assessing environmental and clinical outcomes of the decontamination methods. The available literature was consistent in the observation that automated disinfection by any of the four WRD devices reduced the environmental and the clinical outcomes considerably. All WRD types were effective against various micro-organisms, reducing both environmental microbial contamination of patient rooms and infection rates among patients occupying those rooms.

No distinct variation or classification in in situ effectiveness of the WRD systems can be made, which contrasts to in vitro efficacy. When subjectively comparing the in vitro efficacies, H_2_O_2_ vapour achieves the highest microbial reduction of the four WRD types. This observation is also described is several other reviews and comparative studies [1, 12, 13, 31]. The in situ efficacy of the four WRD systems as described by the (significant) ranges of effect of environmental and clinical outcomes did not considerably vary between systems. Moreover, due to the variability in the included methodologies of included articles and the variability in the available information for specific WRD types and micro-organisms, firm conclusions regarding the differences of in situ efficacy of the four systems may not be drawn. The observation that all WRD systems lead to a reduction in both environmental outcomes as clinical outcomes is coherent with those made in other reviews [109–112].

Automated whole room disinfection systems did not completely remove the pathogens from the environment in all of the included studies, but did lead to a considerable decrease in the microbial load. Although a complete reduction of microbial contamination would be the most ideal scenario, this considerable decrease already lowers the chance of infection tremendously. Moreover, as a contrast to manual cleaning, the observed reduction in pathogens in a hospital setting is a constant and reliable outcome as it is not dependable of an operator. For manual cleaning and disinfection, the decrease in microbial load is greatly dependent on the individual performing the procedure and is limited by human errors [1–3].

### Considerations for choosing a WRD system

Due to the comparable in situ efficacy of the WRD systems, the assessment of what WRD device is most suitable in a specific healthcare setting may be mostly dependable on practical considerations. These considerations include among else the duration of disinfection, the preparations prior to disinfection, the purchase and operating costs, and the user friendliness of a device.

Even though WRD systems offer an unique opportunity for improved cleaning and disinfection practices, it is important to note that automated disinfection will never reduce the total time needed for the terminal cleaning and disinfection process as it always has to be preceded by (manual) cleaning. The added value of automated WRD lies in the enhancement of the quality of the terminal cleaning and disinfection process. In general, methods making use of UV are faster than methods making use of HP. After disinfection using UV, rooms are directly accessible. On the contrary, when disinfecting with HP, the concentration of HP first has to lower to 1 ppm before a room can be entered safely. Moreover, HP systems also require more preparations prior to disinfection which is labour intensive and time consuming. To prevent leakage of hydrogen peroxide, the rooms need to be sealed off from the external environment by shutting the doors with tape and covering the ventilation system. For UV, the main preparation is to enlarge the contact are and to decrease the shading area as much as possible. As a consequence, HP-based WRD systems may be less suitable for the standard disinfection in healthcare settings that have a high patient turnover, due to the greater duration of disinfection and preparations.

In contrast to UV systems, HP systems are able to disinfect difficult-to-reach areas whilst UV-C systems are limited to exposed surfaces that are reached by UV radiation. H_2_O_2_ vapour systems are superior to aHP systems in this matter as they reach a more homogenous distribution of HP. The applicability of a system may therefore also be determined by the need or urgency to disinfect every inch of a patient room thoroughly. This may for example be dependent on the pathogenicity of micro-organisms or its transmission route.

Regarding costs, devices making use of HP are less expensive in purchase than devices making use of UV radiation. However, user or operation costs are more expensive for HP devices as hydrogen peroxide tanks have to be replaced periodically whilst UV lamps only need changing once every few years; although the frequency depends on the intensity of usage.

To summarize, the choice for the most suitable WRD device cannot be summarized in a one-size-fits-all approach as the most suitable option relates to hospital-specific requirements.

### Importance of manual cleaning

Automated room disinfection must always be preceded by a (manual) cleaning process. Both in vitro and in situ findings report that the effectiveness of WRD systems is limited in the presence of organic soil, such as blood or faeces [12, 15, 18, 29, 44, 57, 60, 88, 98]. Controversially, some in situ studies included in this review observed that also without cleaning, automated WRD (significantly) reduced microbial contamination [17, 25, 60–62, 69, 100]. However, as these studies were executed in a real-life hospital setting, it is expected that the rooms were not extremely soiled. They therefore do not invalidate the importance of cleaning before automated disinfection.

### Importance of improved cleaning and disinfection

The importance of an improved cleaning and disinfection process, of which the replacement of manual disinfection by automated disinfection is an example, is highlighted by the current problem with antimicrobial resistance. The world health organization (WHO) has declared that antimicrobial resistance is one of the ten most pressing public health threats. The WHO stated that one of the factors contributing to the spread of microbes, of which some resistant to antimicrobial treatment, is inadequate infection prevention [113]. By improving microbial decontamination and therefore limiting the spread of micro-organisms, WRD could contribute to the constraint of antimicrobial resistance. This effect could possibly be extra pronounced when the devices are implemented in countries with high antimicrobial resistance (in which implementation is feasible). A study researching antibiotic resistance in 41 countries identified that low and middle income countries show higher antimicrobial rates than high-income counties with the drug resistance index being in India [114]. Within Europe, the most recent data as published by the European Centre for Disease Prevention and Control (ECDC) in 2019 observed that most resistant isolates were detected in Greece, Romania, Italy and Bulgaria [115].

### Strength and limitations

A main strength of this review is its systematic approach in reviewing literature. Moreover, it is presumed that this review is the first to give a simultaneous overview of both in situ and in vitro efficacy, as well as practical considerations of the WRD systems. Moreover, besides reporting solely the environmental outcomes, this review also gave insight into the effect of WRD systems on both environmental and clinical outcomes.

The main limitation of this review is the substantial variation in methodology and outcome measures of the included studies. Moreover, the availability of data was also limited to only a section of the preselected micro-organisms. As a consequence, no specific conclusions can be made regarding the in situ efficacy of the WRD devices. Nonetheless, a general conclusion that can be drawn is that all ARD systems are effective in reducing the pathogenic load in a hospital setting. Another limitation of this review is that no distinction was made in the clinical outcome measure between microbial colonization (acquisition) infection rates and clinical infection rates. Stratification could not be performed as this would lead to a too limited number of articles. A final limitation of this review is that most included studies are performed in a high-income or resource countries. As the infection rates greatly vary between high and middle-to-low income countries, the results of this review might not directly relatable to low or middle income counties.

### Recommendations

Due to the variability in research methodologies of the included articles, this review can only generally conclude that all WRD systems are effective in a healthcare setting. It is however not possible to conclude what system is most effective in situ. As such data could influence the choice for the best fitting WRD device in a specific healthcare setting, we recommend the initiation of a study that directly compares the four types of WRD devices regarding their in situ efficacy. If such a study uses a standardized protocol assessing preferably both environmental and clinical outcome measures for all WRD devices and for a range of pathogens, it would be possible to compare the in situ efficacy of the different types of WRD devices. Moreover, we recommend to base the choice for the best fitting automated WRD device in a healthcare setting as much on other practical considerations, such as disinfection time and preparation of a patient room, as on efficacy or even more. These practical considerations will eventually determine how well a devices performs with a healthcare setting.

## Conclusion

In conclusion, despite the large variation in the included studies, all automated WRD systems (aHP, H_2_O_2_ vapour, UV-C and PX-UV) are effective in reducing the amount of pathogens present in a hospital environment. Due to the comparable in situ effectiveness of the WRD systems, the assessment of what WRD device is most suitable in a specific healthcare setting may mostly depend on practical considerations.

## Supplementary Information

**Additional file 1: Table S1.** The in situ efficacy of aerosolized hydrogen peroxide (aHP) as expressed in environmental outcomes. **Table S2.** The in situ efficacy of aerosolized hydrogen peroxide (aHP) as expressed in clinical outcomes. **Table S3.** The in situ efficacy of H_2_O_2_ vapour as expressed in environmental outcomes. **Table S4.** The in situ efficacy of H_2_O_2_ vapour as expressed in clinical outcomes. **Table S5.** The in situ efficacy of ultraviolet C (UV-C) as expressed in environmental outcomes. **Table S6.** The in situ efficacy of ultraviolet C (UV-C) as expressed in clinical outcomes. **Table S7.** The in situ efficacy of pulsed xenon ultraviolet (PX-UV) as expressed in environmental outcomes. **Table S8.** The in situ efficacy of pulsed xenon ultraviolet (PX-UV) as expressed in clinical outcomes.

## Data Availability

The articles included in this literature review were all retrieved from PubMed and can be found in the citations.

## Abbreviations

*A. baumannii*: *Acinetobacter baumannii*
aHP: Aerosolized hydrogen peroxide; whole room disinfection system making use of a 5–6% hydrogen peroxide solution
*C. auris*: *Candida auris*
*C. difficile*: *Clostridium difficile*
CPE: Carbapenemase-producing enterobacteriaceae
CFU: Colony-forming units
ESBL: Extended spectrum beta-lactamase
HAI: Hospital-acquired infection
H_2_O_2_ vapour: Whole room disinfection system making use of a 30–35% hydrogen peroxide solution
HP: Hydrogen peroxide
HPV: A type of H_2_O_2_ vapour device produced by the manufacturer Bioquell
MDRO: Multidrug resistant micro-organism
MRSA: Methicillin-resistant *Staphylococcus aureus*
NoV: Norovirus
PX-UV: Pulsed xenon ultraviolet; whole room disinfection system making use of pulsing UV radiation
UV: Ultraviolet
UV-C: Ultraviolet C; whole room disinfection making use of UV radiation within the UV-C spectrum
VRE: Vancomycin-resistant enterococcus
WRD: Whole room disinfection
