# Supplementary material for: The in situ efficacy of whole room disinfection devices: a literature review with practical recommendations for implementation
Source: Antimicrob Resist Infect Control. 2022 Dec 5;11:149. doi: 10.1186/s13756-022-01183-y (PMC9724435; doi:10.1186/s13756-022-01183-y)
Supplement: Supplementary file 1 — Additional file 1: Table S1. The in situ efficacy of aerosolized hydrogen peroxide (aHP) as expressed in environmental outcomes. Table S2. The in situ efficacy of aerosolized hydrogen peroxide (aHP) as expressed in clinical outcomes. Table S3. The in situ efficacy of H2O2 vapour as expressed in environmental outcomes. Table S4. The in situ efficacy of H2O2 vapour as expressed in clinical outcomes. Table S5. The in situ efficacy of ultraviolet C (UV-C) as expressed in environmental outcomes. Table S6. The in situ efficacy of ultraviolet C (UV-C) as expressed in clinical outcomes. Table S7. The in situ efficacy of pulsed xenon ultraviolet (PX-UV) as expressed in environmental outcomes. Table S8. The in situ efficacy of pulsed xenon ultraviolet (PX-UV) as expressed in clinical outcomes. [file 13756_2022_1183_MOESM1_ESM.docx]

**Additional tables 1-8: overview of included articles**

These tables do not have to be placed in the article. If possible, we would like to include these tables in an additional file.

*Additional Table 1: the in situ efficacy of aerosolized hydrogen peroxide (aHP) as expressed in environmental outcomes.*

| Micro-organism | Study | Country | Care institution | Pre-cleaning | N(%) or CFU of positive cultures pre-intervention | N(%) or CFU of positive cultures post-cleaning | N(%) or CFU of positive cultures post-disinfection | Change in outcome | Statistical significance | aHP device | Type (if noted) |
| --- | --- | --- | --- | --- | --- | --- | --- | --- | --- | --- | --- |
| Norovirus |  |  |  |  |  |  |  |  |  |  |  |
| *Acinetobacter* | Cobrado et al, 2018 ([24](#_ENREF_24)) | Portugal | Burns unit | Yes, with QAC | 30 CFUs/cm^2^ floor  10 CFUs/cm^2^ television  <5 CFUs/cm^2^ bed and bathroom | NA | 0 CFUs/cm^2^ at all locations | -100% | NA | Glosair^TM^ | MDR Acinetobacter |
|  | Lerner et al, 2020 ([25](#_ENREF_25)) | Israel | Tertiary-care hospital | No | 59/74 sites (80%) | NA | 12/68 sites (18%) | -78% | NA | Glosair^TM^ | CRAB |
| CPE |  |  |  |  |  |  |  |  |  |  |  |
| ESBL |  |  |  |  |  |  |  |  |  |  |  |
| MRSA | Bartels et al., 2008 ([116](#_ENREF_116)) | Netherlands | Hospital | No | 8/28 chairs (29%) | NA | 1/28 chairs (4%) | -86% | NA | Sterinis® |  |
|  | McKnew et al, 2021 ([26](#_ENREF_26)) | Australia | Burns unit | Yes, with detergent | 65/309 sites (21%) | 20/309 sites (6.5%) | 18/309 sites (5.8%) | ^1-2^: -69.2%  ^1-3:^ -72%  ^2-3:^ -10% | NA | Deprox^TM^ |  |
|  | Taneja et al., 2011([27](#_ENREF_27)) | India | Medical Education and Research emergency complex | Yes, with detergent | 379 colonies | NA | 0 colonies | -100% | NA | Fogmaster ULV 7401 |  |
|  | Mitchell et al., 2014 ([28](#_ENREF_28)) | Australia | Public hospital | Yes, with detergent | NA | 473/1917 rooms (24.7%) | 322/1712 rooms (18.8%) | -23.8% | P<0.001* | Nocospray® |  |
| VRE | McKnew et al, 2021 ([26](#_ENREF_26)) | Australia | Burns unit | Yes, with detergent | 10/310 sites (3.2%) | 2/310 sites (0.6%) | 0/310 sites (0%) | ^1-2:^ -80%  ^1-3:^ -100%  ^2-3:^ -100% | NA | Deprox^TM^ |  |
| *Clostridium difficile* | Shapey et al, 2008 ([15](#_ENREF_15)) | UK | Elderly care ward | Yes, with detergent | NA | 48/203 sites (24%)  138 CFU | 7/203 sites (3%)  8 CFU | -87.5%  -94% | P<0.0001*  P<0.0001* | Sterinis® |  |
|  | Barbut et al., 2009 ([30](#_ENREF_30)) | France | Two hospitals | NA | 34/180 sites (19%) | NA | 4/180 sites (2%) | -91% | P<0.005* | Not described |  |
|  | Yui et al., 2017 ([29](#_ENREF_29)) | UK | Tertiary referral centre teaching hospital | Yes, with detergent | 131/572 sites (23%) | 105/959 sites (10.6%) | 43/967 sites (4.4%) | ^1-2:^ -52.2%  ^1-3:^ -80.6%  ^2-3:^ -59.4% | NA | Deprox^TM^ |  |
| *Candida auris* |  |  |  |  |  |  |  |  |  |  |  |

*Statistically significant findings (p<0.05) are indicated with an asterisk (*).*

*Additional Table 2: the in situ efficacy of aerosolized hydrogen peroxide (aHP) as expressed in clinical outcomes.*

| **Micro-organism** | **Study** | **Country** | **Care institution** | **Pre-cleaning** | **Incidence rate pre-intervention per 1000 PD** | **Incidence rate post-intervention per 1000 PD** | **Change in outcome** | **Statistical Significance** | **aHP device** | **Remark** |
| --- | --- | --- | --- | --- | --- | --- | --- | --- | --- | --- |
| Norovirus |  |  |  |  |  |  |  |  |  |  |
| *Acinetobacter* |  |  |  |  |  |  |  |  |  |  |
| CPE |  |  |  |  |  |  |  |  |  |  |
| ESBL |  |  |  |  |  |  |  |  |  |  |
| MRSA | Mitchell et al., 2014 ([28](#_ENREF_28)) | Australia | Public hospital | Yes, with detergent | 0.9 | 0.53 | -41.1% | P<0.001* | Nocospray® |  |
| VRE |  |  |  |  |  |  |  |  |  |  |
| *Clostridium difficile* |  |  |  |  |  |  |  |  |  |  |
| *Candida auris* |  |  |  |  |  |  |  |  |  |  |

*Statistically significant findings (p<0.05) are indicated with an asterisk (*).*

*Additional Table 3: the in situ efficacy of H_2_O_2_ vapour as expressed in environmental outcomes.*

| **Micro-organism** | **Study** | **Country** | **Care institution** | **Pre-cleaning** | **N(%) or CFU of positive cultures pre-intervention** | **N(%) or CFU of positive cultures post-cleaning** | **N(%) or CFU of positive cultures post-disinfection** | **Change in outcome** | **Statistical Significance** | **HPV device** | **Remark** |
| --- | --- | --- | --- | --- | --- | --- | --- | --- | --- | --- | --- |
| Norovirus |  |  |  |  |  |  |  |  |  |  |  |
| *Acinetobacter* | Otter et al., 2010 ([49](#_ENREF_49)) | Netherlands | ICU | NA | 10/21 rooms (47.6%) | NA | 0/21 rooms | -100% (10/10) | NA | Bioquell | Acinetobacter spp |
|  | Manian et al., 2011 ([6](#_ENREF_6)) | US | Hospital | Yes, with bleach | NA | 51/312 rooms (16.4%) | 6/312 rooms (4.5%) | -73% | P=0.04* | Bioquell | *A. baumannii* |
| CPE |  |  |  |  |  |  |  |  |  |  |  |
| ESBL |  |  |  |  |  |  |  |  |  |  |  |
| MRSA | Otter et al., 2007 ([50](#_ENREF_50)) | UK | Hospital | Yes, with detergent (QAC) | 18/30 sites^1^ (60%) | 12/30 sites(40%)^2^ | 1/30 sites (3.3%)^3^ | ^1-2:^ -33.3%  ^1-3:^ -94.4%  ^2-3:^ -91.7% | NA | Bioquell |  |
|  | Dryden et al., 2008 ([51](#_ENREF_51)) | US | Surgical ward | Not mentioned | 8/29 sites (27.6%) | NA | 1/29 sites (3.4%) | -87.7% | NA | Bioquell |  |
|  | Jeanes et al., 2005 ([52](#_ENREF_52)) | UK | Surgical ward | Yes, with detergent (chlorine) | 10/28 sites (35.7%) | 2/15 sites (13.3%) | 0/50 sites (0%) | ^1-2:^ -62.7%  ^1-3:^ -100% (10/10)  ^2-3:^ -100% (2/2) | NA | Bioquell |  |
|  | French et al., 2004 ([4](#_ENREF_4)) | UK | Hospital | Yes, with detergent | 61/85 sites (72%) | NA | 1/85 sites (1.2%) | -98.3% | NA | Bioquell |  |
|  | Hardy et al., 2007 ([53](#_ENREF_53)) | UK | ICU | Yes, with detergent | NA | 5/29 sites (17.2%) | 0/29 sites (100%) | -100% (5/5) | NA | Bioquell |  |
|  | Manian et al., 2011 ([6](#_ENREF_6)) | US | Tertiary care hospital | Yes, with detergent (bleach) | NA | 44 rooms (14.1%)  108 sites (1.9%) | 2 rooms (1.5%)  2 sites (0.75%) | -95.5%  -98.1% | NA  P=0.04* | Bioquell |  |
|  | Passaretti et al., 2013 ([41](#_ENREF_41)) | US | Tertiary hospital | Yes, with QAC | NA | 3/170 rooms (1.8%) | 8/397 rooms (2.0%) | +11.1% | P=1.00 | Bioquell |  |
| *VRE* | Passaretti et al., 2013 ([41](#_ENREF_41)) | US | Tertiary hospital | Yes, with QAC | NA | 16/170 rooms (9.4%) | 35/397 rooms (8.8%) | -6.4% | P=0.84 | Bioquell |  |
|  | Otter et al., 2007 ([50](#_ENREF_50)) | UK | Hospital | Yes, with detergent (QAC) | 1/15 sites (6.7%) | 1/15 sites (6.7%) | 0/15 sites (0%) | -100%  (1/1) | NA | Bioquell |  |
| *C. difficile* | Passaretti et al., 2013 ([41](#_ENREF_41)) | US | Tertiary hospital | Yes, with QAC | NA | 1/170 rooms (0.6%) | 0/397 rooms (0%) | -100%  (1/1) | P=0.30 | Bioquell |  |
|  | Boyce et al., 2008 ([54](#_ENREF_54)) | US | Hospital | Yes, with detergent | NA | 11/43 sites (25.6%) | 0/43 sites (0%) | -100%  (11/11) | P<0.001* | Bioquell |  |
| *Candida auris* |  |  |  |  |  |  |  |  |  |  |  |

*Statistically significant findings (p<0.05) are indicated with an asterisk (*).*

*Additional Table 4: the in situ efficacy of H_2_O_2_ vapour as expressed in clinical outcomes.*

| **Micro-organism** | **Study** | **Country** | **Care institution** | **Pre-cleaning** | **Incidence rate pre-intervention per 1000 PD** | **Incidence rate post-intervention per 1000 PD** | **Change in outcome** | **Statistical Significance** | **HPV device** | **Remark** |
| --- | --- | --- | --- | --- | --- | --- | --- | --- | --- | --- |
| Norovirus |  |  |  |  |  |  |  |  |  |  |
| *Acinetobacter* |  |  |  |  |  |  |  |  |  |  |
| CPE |  |  |  |  |  |  |  |  |  |  |
| ESBL |  |  |  |  |  |  |  |  |  |  |
| MRSA | Passaretti et al., 2013 ([41](#_ENREF_41)) | US | Tertiary hospital | Yes, with QAC | 3.7 | 1.2 | -68% | P=0.30 | Bioquell |  |
| VRE | Passaretti et al., 2013 ([41](#_ENREF_41)) | US | Tertiary hospital | Yes, with QAC | 11.6 | 2.4 | -79.3% | P<0.01* | Bioquell |  |
| *C. difficile* | Passaretti et al., 2013 ([41](#_ENREF_41)) | US | Tertiary hospital | Yes, with QAC | 2.7 | 1.0 | -63% | P=0.19 | Bioquell |  |
|  | Boyce et al., 2008 ([54](#_ENREF_54)) | US | Hospital; affected wards | Yes, with detergent | 2.28 | 1.28 | -43.9% | P=0.047* | Bioquell |  |
|  | Boyce et al., 2008 ([54](#_ENREF_54)) | US | Hospital; entire hospital | Yes, with detergent | 1.36 | 0.84 | -38.2% | P=0.26 | Bioquell |  |
|  | McCord et al., 2016 ([40](#_ENREF_40)) | US | Hospital | Yes, with detergent (sporicidal cleaner) | 1.0 | 0.4 | -60% | P<0.001* | Bioquell |  |
|  | Manian et al., 2013 ([39](#_ENREF_39)) | US | Community hospital | Yes, with detergent (bleach) | 0.88 | 0.55 | -37% | P<0.0001* | Bioquell |  |
| *Candida auris* |  |  |  |  |  |  |  |  |  |  |

*Statistically significant findings (p<0.05) are indicated with an asterisk (*).*

*Additional Table 5: the in situ efficacy of ultraviolet C (UV-C) as expressed in environmental outcomes.*

| Micro-organism | Study | Country | Care institution | Pre-cleaning | N(%) or CFU of positive cultures pre-intervention | N(%) or CFU of positive cultures post-cleaning | N(%) or CFU of positive cultures post-disinfection | Change in outcome | Statistical Significance | UV-C device | Remark |
| --- | --- | --- | --- | --- | --- | --- | --- | --- | --- | --- | --- |
| Norovirus |  |  |  |  |  |  |  |  |  |  |  |
| *Acinetobacter* | Anderson et al., 2013 ([76](#_ENREF_76)) | US | Two tertiary care hospitals | NA | 52 CFU | NA | 1 CFU | -98% | P=0.25 | Tru-D® |  |
|  | Rutala et al., 2018 ([77](#_ENREF_77)) | US | Tertiary care hospital | Yes, with QAC  Yes, with bleach | NA | 8.95 CFU  0.39 CFU | 0.18 CFU  0.25 CFU | -98%  -35.9% | P=0.017*  NA | NA | MDR acinetobacter |
| ESBL |  |  |  |  |  |  |  |  |  |  |  |
| MRSA | Nerandzic et al., 2010 ([60](#_ENREF_60)) | US | Acute care hospital | Not cleaned before decontamination | 28/261 sites (10.7%)  0.02 CFU/cm^2^ | NA | 2/261 sites (0.8%)  0.0042 CFU/cm^2^ | -93%  -79% | P<0.01*  P<0.001* | Tru-D® |  |
|  | Rutala et al., 2010 ([61](#_ENREF_61)) | US | Acute care hospital | Not cleaned | 81/400 sites (20.3%)  384 CFU | NA | 2/400 sites  (0.5%)  19 CFU | -97.5%  -95% | P<0.001*  P<0.001* | Tru-D® |  |
|  | Yang et al., 2019 ([62](#_ENREF_62)) | Taiwan | Acute medical care centre | Not cleaned | 35 CFU  15/20 sites (75%) | NA  NA | 0 CFU  4/20 sites (20%) | -100%  -73.3% | P=0.0005*  NA | Hyper Light P3 |  |
|  | Wong et al., 2016 ([78](#_ENREF_78)) | US | Academic teaching hospital | Yes, with detergent (accelerated hydrogen peroxide) | 21/61 rooms (34.4%)  50/360 sites (13.9%) | 17/61 rooms (27.9%)  21/360 sites (5.8%) | 2/61 rooms (3.3%)  2/360 sites (0.55%) | ^1-2:^- 19%  ^1-3:^ -90.5%  ^2-3:^ -88.2%  ^1-2:^ -58%  ^1-3:^ -96%  ^2-3:^ -90.5% | ^1-2:^ P=0.502 ^2-3:^ P=0.00003*  ^1-2:^  P<0.001* &  ^2-3:^  P <0.001* | Tru-D & R-D Rapid disinfector |  |
|  | Mustapha et al., 2018 ([79](#_ENREF_79)) | US | Veteran affairs hospital | Yes, with detergent | 9/27 rooms (33%) | 3/27 rooms (11%) | 1/27 rooms (1%) | ^1-2:^ -66.7%  ^1-3:^ -88.9%  ^2-3:^ -66.7% | ^1-2:^  P<0.01* &  ^2-3:^  P>0.05 | Clorox® |  |
|  | Rutala et al., 2018 ([77](#_ENREF_77)) | US | 3 study hospitals | Yes, with detergent (QAC or bleach) | NA | 8.52 CFU  4.39 CFU | 0.11 CFU  0.85 CFU | -98.7%  -80.6% | P=0.032*  NA | NA |  |
| VRE | Nerandzic et al., 2010 ([60](#_ENREF_60)) | US | Acute care hospital | Not cleaned before decontamination | 7/261 sites (2.7%)  0.003 CFU/cm^2^ | NA  NA | 1/261 sites (0.38%)  0 CFU/cm^2^ | -93%  -100% | P=0.007*  P<0.001* | Tru-D® |  |
|  | Rutala et al., 2010 ([61](#_ENREF_61)) | US | Acute care hospital | Not cleaned | 384 CFU | NA | 19 CFU | -95.1% | P<0.001* | Tru-D® |  |
|  | Yang et al., 2019 ([62](#_ENREF_62)) | Taiwan | Acute medical care centre | Not cleaned | 35 CFU  15/20 sites (75%) | NA  NA | 0 CFU  4/20 sites (20%) | -100%  -73.3% | P=0.0005*  NA | Hyper Light P3 |  |
|  | Wong et al., 2016 ([78](#_ENREF_78)) | US | Academic teaching hospital | Yes, with detergent (accelerated hydrogen peroxide) | 18/61 rooms (29.5%)  41/360 sites (11.4%) | 18/61 rooms (29.5)`  25/360 sites (6.9%) | 3/61 rooms (4.9%)  3/360 sites (0.83%) | -83.3%  ^1-2:^ -39%  ^1-3:^ -92.7%  ^2-3:^ -88% | P=0.0003*  P<0.01* | Tru-D® & R-D Rapid disinfector |  |
|  | Anderson et al., 2013 ([76](#_ENREF_76)) | US | Two tertiary care hospitals | Unknown | 712 CFU | NA | 15 CFU | -97.9% | P<0.0001* | Tru-D® |  |
|  | Rutala et al., 2018 ([77](#_ENREF_77)) | US | 3 study hospitals | Yes, with detergent (QAC or bleach) | NA | 39.57 CFU  2.43 CFU | 0.21 CFU  1.9 CFU | -99.5%  -21.8% | P=0.034*  NA | NA |  |
| *C. Difficile* | Nerandzic et al., 2010 ([60](#_ENREF_60)) | US | Acute care hospital | Not cleaned before decontamination | 9/261 sites (3.4%)  0.01 CFU/cm^2^ | NA  NA | 1/261 sites (0.38%)  0.0004 CFU/cm^2^ | -80%  -96% | P=0.02*  P<0.001* | Tru-D® |  |
|  | Wong et al., 2016 ([78](#_ENREF_78)) | US | Academic teaching hospital | Yes, with detergent (accelerated hydrogen peroxide) | 7/22 rooms (31.8%)  9/125 sites (7.2%) | 5/22 rooms (22.7%)  5/125 sites (4%) | 0/22 rooms(0%)  0/125 sites (0%) | ^1-2:^ -28.6%  ^1-3:^ -100%  ^2-3:^ -100%  ^1-2:^ -44.4%  ^1-3:^ -100%  ^2-3:^ -100% | ^1-2:^ P=0.617 ^2-3:^ P=0.0736  ^1-2:^ P =0.343 ^2-3:^ P<0.001* | Tru-D® & R-D Rapid disinfector |  |
|  | Anderson et al., 2013 ([76](#_ENREF_76)) | US | Two tertiary care hospitals | Unknown | 724 CFU | NA | 51 CFU | -93% | P=0.25 | Tru-D |  |
|  | Liscynesky et al., 2017 ([80](#_ENREF_80)) | UK | Comprehensive cancer centre | Yes, with detergent (bleach) | NA | 32/238 sites (13%) | 1/238 sites (0.4%) | -96.9% | NA | Surfacide UV-C |  |
|  | Rutala et al., 2018 ([77](#_ENREF_77)) | US | 3 study hospitals | Yes, with detergent (QAC or bleach) | NA | 3.76 CFU  4.48 CFU | 2.86 CFU  3.25 CFU | -23.9%  -27.5% | NA  NA | NA |  |
|  | Mustapha et al., 2018 ([79](#_ENREF_79)) | US | Veteran affairs hospital | Yes, with detergent | 9/27 rooms (33%) | 0/27 rooms | 0/27 rooms | -100% by cleaning (9/9) | P<0.01* (for cleaning) | Clorox® |  |
| *Candida auris* | Mustapha et al., 2018 ([79](#_ENREF_79)) | US | Veteran affairs hospital | Yes, with detergent | 8/27 rooms (30%) | 0/27 rooms | 0/27 rooms | -100% by cleaning (9/9) | P<0.01* (for cleaning) | Clorox® |  |

*Statistically significant findings (p<0.05) are indicated with an asterisk (*)*

*Additional Table 6: the in situ efficacy of ultraviolet C (UV-C) as expressed in clinical outcomes.*

| **Micro-organism** | **Study** | **Country** | **Care institution** | **Pre-cleaning** | **Incidence rate pre-intervention per 1000 PD** | **Incidence rate post-intervention per 1000 PD** | **Change in outcome** | **Statistical Significance** | **UV-C device** | **Remark** |
| --- | --- | --- | --- | --- | --- | --- | --- | --- | --- | --- |
| Norovirus |  |  |  |  |  |  |  |  |  |  |
| *Acinetobacter* | Anderson et al., 2018 ([81](#_ENREF_81)) | US | 9 hospitals | Yes, with detergent (QAC) | 0.018 | 0.008 | -55.5% | NA | TRU-D® | MDR Acinetobacter spp |
|  | Raggi et al., 2018 ([82](#_ENREF_82)) | US | Community hospital | Yes, with detergent | 0.34 | 0.16 | -53.1% | P=0.03* | Skrypton IPT UV-C | Acinetobacter baumannii |
|  | Napolitano et al., 2015 ([83](#_ENREF_83)) | US | Community hospital | Yes, with detergent | 0.39 | 0.11 | -71.8% | P=0.005* | IRIS ^TM^ 3200m | Acinetobacter baumannii |
| CPE | Raggi et al., 2018 ([82](#_ENREF_82)) | US | Community hospital | Yes, with detergent | 1.16 | 1.22 | +7.5% | P=0.36 | Skrypton IPT UV-C | K. Pneumoniae |
|  | Napolitano et al., 2015 ([83](#_ENREF_83)) | US | Community hospital | Yes, with detergent | 0.44 | 0 | -100% | P<0.001* | IRIS ^TM^ 3200m | K. Pneumoniae |
| ESBL | Raggi et al., 2018 ([82](#_ENREF_82)) | US | Community hospital | Yes, with detergent | 1.29 | 1.16 | -12.9% | P=0.22 | Skryton IPT UV-C | Pseudomonas aeruginosa |
| MRSA | Raggi et al., 2018 ([82](#_ENREF_82)) | US | Community hospital | Yes, with detergent | 1.42 | 0.98 | -30.8% | P=0.02* | Skryton IPT UV-C |  |
|  | Napolitano et al., 2015 ([83](#_ENREF_83)) | US | Community hospital | Yes, with detergent | 0.39 | 0.38 | -1.2% | P=1 | IRIS^TM^ 3200m |  |
|  | Anderson et al., 2018 ([81](#_ENREF_81)) | US | 9 hospitals | Yes, with detergent (QAC) | 0.57 | 0.63 | +9.5% | P=0.42 | Tru-D® |  |
| VRE | Anderson et al., 2018 ([81](#_ENREF_81)) | US | 9 hospitals | Yes, with detergent (QAC) | 0.324 | 0.323 | -0.3% | P=0.048* | Tru-D® |  |
|  | Raggi et al., 2018 ([82](#_ENREF_82)) | US | Community hospital | Yes, with detergent | 0.68 | 0.45 | -33.8% | P=0.005* | Skrypton IPT UV-C |  |
|  | Napolitano et al., 2015 ([83](#_ENREF_83)) | US | Community hospital | Yes, with detergent | 1 | 0.88 | -12.3% | P=0.14 | IRIS ^TM^ 3200m |  |
| *C. Difficile* | Anderson et al., 2018 ([81](#_ENREF_81)) | US | 9 hospitals | Yes, with detergent (QAC) | 1.01 | 0.913 | -9.6% | P=0.031* | Tru-D® |  |
|  | Pegues et al., 2017 ([84](#_ENREF_84)) | US | Haematology-oncology unit | Yes, with detergent | 3.03 | 2.29 | -25% | P=0.03* | Optimum-UV® |  |
|  | McMullen et al., 2020 ([85](#_ENREF_85)) | US | 3 hospitals | Yes, with detergent | 0.69  0.34  0.96 | 0.64  0.46  1.18 | -7.2%  +26.1%  +18.6% | P=0.53  P=0.22  P=0.22 | Surfacide UV-C |  |
|  | Liscynesky et al., 2017 ([80](#_ENREF_80)) | UK | Comprehensive cancer centre | Yes, with detergent (bleach) | 1.29 | 1.14 | -11.6% | NA | Surfacide UV-C |  |
|  | Napolitano et al., 2015 ([83](#_ENREF_83)) | US | Community hospital | Yes, with detergent | 1.23 | 0.66 | -46.2% | P<0.001* | IRIS ^TM^ 3200m |  |
| *Candida auris* |  |  |  |  |  |  |  |  |  |  |

*Statistically significant findings (p<0.05) are indicated with an asterisk (*)*

*Additional Table 7: the in situ efficacy of pulsed xenon ultraviolet (PX-UV) as expressed in environmental outcomes.*

| **Micro-organism** | **Study** | **Country** | **Care institution** | **Pre-cleaning** | **N(%) or CFU of positive cultures pre-intervention** | **N(%) or CFU of positive cultures post-cleaning** | **N(%) or CFU of positive cultures post-disinfection** | **Change in outcome** | **Statistical Significance** | **Machine** | **Remark** |
| --- | --- | --- | --- | --- | --- | --- | --- | --- | --- | --- | --- |
| Norovirus |  |  |  |  |  |  |  |  |  |  |  |
| *Acinetobacter* |  |  |  |  |  |  |  |  |  |  |  |
| CPE |  |  |  |  |  |  |  |  |  |  |  |
| ESBL |  |  |  |  |  |  |  |  |  |  |  |
| MRSA | Nerandzic et al., 2015 ([69](#_ENREF_69)) | US | 2 acute care hospitals | Without cleaning | 11/112 sites (10%)  9 CFU | NA | 1/112 sites (0.9%)  2 CFU | -90.9%  -77.8% | P<0.01* | Xenex® |  |
|  | Nerandzic et al., 2015 ([69](#_ENREF_69)) | US | 2 acute care hospitals | With cleaning (chlorine) | 11/113 sites (10%)  96 CFU | NA | 2/113 sites (2%)  12 CFU | -81.8%  -87.5% | P<0.01* | Xenex® |  |
|  | Zeber et al., 2018 ([97](#_ENREF_97)) | US | 4 Veterans affairs hospitals | Yes, with disinfectant | NA | 397 colonies per room | 98 colonies per room | -75.3% | NA | Xenex® |  |
|  | Kitagawa et al., 2020 ([102](#_ENREF_102)) | Japan | Hospital | Yes | NA | 1.1 CFU | 0.3 CFU | -72.7% | P<0.001* | Xenex® |  |
|  | Jinadatha et al., 2015 ([100](#_ENREF_100)) | US | Tertiary care hospital | No | 393 colonies | NA | 100 colonies | -74.6% | P<0.01* | Xenex® |  |
|  | Jinadatha et al., 2014 ([101](#_ENREF_101)) | US | Acute care hospital | Yes | 108 colonies^1^ | 11 colonies^2^ | 1 colony^3^ | ^1-2:^ -89.8%  ^1-3:^ -99.1%  ^2-3:^ -90.9% | NA | Xenex® |  |
| VRE | Nerandzic et al., 2015 ([69](#_ENREF_69)) | US | 2 acute care hospitals | Without cleaning | 4/112 sites (3.6%)  21 CFU | NA | 0/112 sites (0%)  0 CFU | -100% (4/4)  -100% | P<0.01*  P<0.01* | Xenex® |  |
|  | Nerandzic et al., 2015 ([69](#_ENREF_69)) | US | 2 acute care hospitals | With cleaning (chlorine) | 4/113 sites (3.5%)  12 CFU | NA | 1/113 sites (0.9%)  1 CFU | -75%  -91.7% | P<0.01*  P<0.01* | Xenex® |  |
|  | Beal et al., 2016 ([91](#_ENREF_91)) | UK | Clinical haematology unit of teaching hospital | Yes, with detergent | NA  35.5 CFU^1^ | 26/80 sites (32.5%)  4 CFU^2^ | 16/80 sites (20%)  2 CFU^3^ | -38%  ^1-2:^ -88.7%  ^1-3:^ -94.4%  ^2-3:^ -50% | P=0.072  NA | Xenex® |  |
|  | Stibich et al., 2011 ([103](#_ENREF_103)) | US | Comprehensive cancer centre | Yes, with detergent | 17/75 sites (23.3%)^1^  33 CFU/cm^2, 1^ | 4/91 sites (8.2%)^2^  27.4 CFU/cm^2, 2^ | 0/75 sites (0%)^3^  1.2 CFU/cm^2, 3^ | ^1-2:^ -80.6%  ^1-3:^ -100% (17/17)  ^2-3:^ -100% (4/4)  ^1-2:^ -17.0%  ^1-3:^ -96.4%  ^2-3:^ -95.6% | P<0.0001* for each step | Xenex® |  |
|  | Kitagawa et al., 2021 ([104](#_ENREF_104)) | Japan | Hospital | Yes | 22/60 sites (37%) | 14/60 sites (23%) | 0/60 sites (0%) | ^1-2:^ -36.4%  ^1-3:^ -100% (22/22)  ^2-3:^ -100% (14/14) | P<0.001* | Xenex® |  |
| *C. difficile* | Nerandzic et al., 2015 ([69](#_ENREF_69)) | US | 2 acute care hospitals | Without cleaning | 22/113 sites (19%) | NA | 9/113 sites (8%) | -59% | P>0.05 | Xenex® |  |
|  | Nerandzic et al., 2015 ([69](#_ENREF_69)) | US | 2 acute care hospitals | With cleaning (chlorine) | 13/112 sites (12%) | NA | 3/112 sites (3%) | -76.9% | P<0.01* | Xenex® |  |
|  | Kitagawa et al., 2021 ([106](#_ENREF_106)) | Japan | Hospital | Yes | 22/71 sites (31%)  125 CFU | NA | 8/71 sites (11%)  24 CFU | -63.6% | P=0.002*  P=0.001* | Xenex ® |  |
|  | Ghantoji et al., 2015 ([105](#_ENREF_105)) | US | Comprehensive cancer centre | No | 22.97 CFU | NA | 1.19 CFU | -94.8% | P=0.0017* | Xenex® |  |
| *Candida auris* |  |  |  |  |  |  |  |  |  |  |  |

*Statistically significant findings (p<0.05) are indicated with an asterisk (*)*

*Additional Table 8: the in situ efficacy of pulsed xenon ultraviolet (PX-UV) as expressed in clinical outcomes.*

| **Micro-organism** | **Study** | **Country** | **Care institution** | **Pre-cleaning** | **Incidence rate pre-intervention per 1000 PD** | **Incidence rate post-intervention per 1000 PD** | **Reduction** | **Statistical Significance** | **Machine** | **Remark** |
| --- | --- | --- | --- | --- | --- | --- | --- | --- | --- | --- |
| Norovirus |  |  |  |  |  |  |  |  |  |  |
| *Acinetobacter* | Morikane et al., 2020 ([89](#_ENREF_89)) | Japan | Tertiary referral hospital | Yes, with detergent | 4.85 | 1.81 | -63% | P<0.001* | Xenex® | Resistant Acinetobacter |
| CPE |  |  |  |  |  |  |  |  |  |  |
| ESBL |  |  |  |  |  |  |  |  |  |  |
| MRSA | Haas et al., 2014 ([107](#_ENREF_107)) | US | Tertiary care academic centre | Yes | 0.45 | 0.33 | -26.7% | P=0.007* | Xenex® |  |
|  | Vianna et al., 2016 ([92](#_ENREF_92)) | US | Community hospital | Yes | 0.34 | 0.41 | +20% | P=0.23 | Xenex ® |  |
|  | Morikane et al., 2020 ([89](#_ENREF_89)) | Japan | Tertiary referral hospital | Yes | 1.38 | 0.99 | -29% | P=0.002* | Xenex® |  |
|  | Kitagawa et al., 2021 ([95](#_ENREF_95)) | Japan | Hospital | Yes, with QAC | 3.56 | 2.21 | -37.9% | P=0.0497* | Xenex® |  |
| VRE | Brite et al., 2018 ([90](#_ENREF_90)) | US | Transplantation unit tertiary care centre | Yes, | 3.0 | 3.7 | +18.9% | P=0.6 | Xenex® |  |
|  | Haas et al., 2014 ([107](#_ENREF_107)) | US | Tertiary care academic centre | Yes | 0.9 | 0.73 | -18.9% | P=0.002* | Xenex® |  |
|  | Vianna et al., 2016 ([92](#_ENREF_92)) | US | Community hospital | Yes, with detergent | 0.34 | 0.17 | -50% | P=0.07 | Xenex® |  |
|  | Sampathkuma et al., 2019 ([93](#_ENREF_93)) | US | Tertiary care hospital | Yes, with detergent (bleach) | 2.6 | 1.23 | -52.7% | NA | Xenex® |  |
| *C. difficile* | Brite et al., 2018 ([90](#_ENREF_90)) | US | Transplantation unit tertiary care centre | Yes, | 1.4 | 1.1 | -21% | P=0.7 | Xenex® |  |
|  | McMullen et al., 2021 ([85](#_ENREF_85)) | US | 2 hospitals | Yes | 0.69  0.96 | 0.64  1.18 | -7.2%  +18.6% | P=0.53  P=0.22 | Xenex® |  |
|  | Haas et al., 2014 ([107](#_ENREF_107)) | US | Tertiary care academic centre | Yes | 0.79 | 0.65 | -17.2% | P=0.02* | Xenex® |  |
|  | Vianna et al., 2016 ([92](#_ENREF_92)) | US | Community hospital | Yes, with detergent | 0.83 | 0.49 | -41% | P=0.01* | Xenex® |  |
|  | Sampathkuma et al., 2019 ([93](#_ENREF_93)) | US | Tertiary care hospital | Yes, with detergent (bleach) | 2.1 | 1.1 | -47.6% | NA | Xenex® |  |
|  | Nagaraja et al., 2015 ([94](#_ENREF_94)) | US | Tertiary care hospital | Yes | 1.06 | 0.83 | -22% | P=0.06 | Xenex® |  |
|  | Levin et al., 2013 ([108](#_ENREF_108)) | US | Acute community care hospital | Yes | 0.95 | 0.45 | -53% | P<0.01* | Xenex® |  |
|  | Attia et al., 2020 ([96](#_ENREF_96)) | US | Tertiary care academic hospital | Yes | 1.57 | 1.61 | +2.5% | NA | NA |  |
| *Candida auris* |  |  |  |  |  |  |  |  |  |  |

*Statistically significant findings (p<0.05) are indicated with an asterisk (*)*
